# Percepta Genomic Sequencing Classifier and decision-making in patients with high-risk lung nodules: a decision impact study

**DOI:** 10.1186/s12890-021-01772-4

**Published:** 2022-01-06

**Authors:** Sonali Sethi, Scott Oh, Alexander Chen, Christina Bellinger, Lori Lofaro, Marla Johnson, Jing Huang, Sangeeta Maruti Bhorade, William Bulman, Giulia C. Kennedy

**Affiliations:** 1 Division of Pulmonary Medicine, Respiratory Institute, Cleveland Clinic, 9500 Euclid Avenue, Mail Code A90, Cleveland, OH 44195 USA; 2 Division of Pulmonary, Critical Care and Sleep Medicine, University of California, Los Angeles, Los Angeles, CA USA; 3 Division of Pulmonary and Critical Care Medicine, Washington University School of Medicine, St. Louis, MO USA; 4 Pulmonary, Critical Care, Allergy and Immunologic Disease, Wake Forest School of Medicine, Winston-Salem, NC USA; 5 Veracyte, Inc., South San Francisco, CA USA

**Keywords:** Lung lesion, Lung cancer, Bronchoscopy, Risk assessment, Physician confidence

## Abstract

**Background:**

Incidental and screening-identified lung nodules are common, and a bronchoscopic evaluation is frequently nondiagnostic. The Percepta Genomic Sequencing Classifier (GSC) is a genomic classifier developed in current and former smokers which can be used for further risk stratification in these patients. Percepta GSC has the capability of up-classifying patients with a pre-bronchoscopy risk that is high (> 60%) to “very high risk” with a positive predictive value of 91.5%. This prospective, randomized decision impact survey was designed to test the hypothesis that an up-classification of risk of malignancy from high to very high will increase the rate of referral for surgical or ablative therapy without additional intervening procedures while increasing physician confidence.

**Methods:**

Data were collected from 37 cases from the Percepta GSC validation cohort in which the pre-bronchoscopy risk of malignancy was high (> 60%), the bronchoscopy was nondiagnostic, and the patient was up-classified to very high risk by Percepta GSC. The cases were randomly presented to U.S pulmonologists in three formats: a pre-post cohort where each case is presented initially without and then with a GSG result, and two independent cohorts where each case is presented either with or without with a GSC result. Physicians were surveyed with respect to subsequent management steps and confidence in that decision.

**Results:**

One hundred and one survey takers provided a total of 1341 evaluations of the 37 patient cases across the three different cohorts. The rate of recommendation for surgical resection was significantly higher in the independent cohort with a GSC result compared to the independent cohort without a GSC result (45% vs. 17%, *p* < 0.001) In the pre-post cross-over cohort, the rate increased from 17 to 56% (*p* < 0.001) following the review of the GSC result. A GSC up-classification from high to very high risk of malignancy increased Pulmonologists’ confidence in decision-making following a nondiagnostic bronchoscopy.

**Conclusions:**

Use of the Percepta GSC classifier will allow more patients with early lung cancer to proceed more rapidly to potentially curative therapy while decreasing unnecessary intervening diagnostic procedures following a nondiagnostic bronchoscopy.

**Supplementary Information:**

The online version contains supplementary material available at 10.1186/s12890-021-01772-4.

## Background

Lung nodules are common findings on low dose CT scans performed for lung cancer screening as well as on CT scans performed for other reasons [1, 2]. Most nodules will not be cancer, but differentiating benign from malignant nodules can be difficult, often leading to costly, invasive procedure in patients without cancer [3]. Moreover, the current diagnostic modalities often fail [4]. Current management guidelines recommend that lung nodules between 8 and 30 mm in size in patients with low surgical risk and high clinical probability of malignancy be considered for primary curative treatment without an intervening biopsy [5]. Likewise, larger lesions (> 30 mm) in at risk individuals have been shown to be highly predictive of cancer [6]. Therefore these patients should also be considered for surgical resection if data suggest that they are likely to have early stage disease. However, studies have shown that physicians frequently opt for more conservative management including further surveillance or other diagnostic procedures in these high-risk patients [7, 8]. For lung lesions at intermediate risk for malignancy [5], or for patients with higher or lower risk where non-surgical tissue sampling is deemed prudent, bronchoscopy is often the modality of choice for determining pathology and directing decision-making around the next management step. Considerable effort has gone into the development of technologies that seek to improve the diagnostic yield of bronchoscopy for suspicious lung lesions, but nondiagnostic procedures remain common [4].

The Percepta Bronchial Genomic Classifier (Percepta, Veracyte, Inc.) was specifically developed to aid in the diagnosis of lung lesions suspected of being lung cancer in the event of a nondiagnostic bronchoscopy [9]. It utilizes the well-established field of injury principle, where patterns of gene expression associated with lung cancer are detectable in cytologically normal-appearing respiratory epithelial cells in current or former smokers [10]. The original classifier was developed using gene microarray technology to capture gene-expression data from benign-appearing bronchial epithelial cells collected from the right mainstem bronchus by cytologic brushing. Applied to patients with an indeterminant lung lesion, the classifier was shown to accurately distinguish those with lung cancer from those with benign disease [11]. The classifier provides a risk re-stratification based on a patient’s pre-test probability of cancer, and the result is a post-test probability of malignancy intended to inform decision-making.

The performance of the classifier was evaluated in two large cohorts totaling 937 patients undergoing bronchoscopy for suspicious lung lesions. 43% of cases were nondiagnostic. In these patients the classifier had an area under the curve (AUC) of 0.74 and 0.78 in the two separate cohorts, with a 91% negative predictive value (NPV) for patients with a pre-test intermediate risk of cancer [9]. The impact of a Percepta result on physician decision-making was also previously studied in a randomized, prospective, decision impact survey study, using the original iteration of the genomic classifier. That study focused on the effect of a low risk (negative) result on reducing a recommendation for further invasive procedures after a nondiagnostic bronchoscopy for nodules at intermediate pre-test risk of malignancy. When physicians had access to clinical information plus a genomic classifier low risk result (compared to clinical information alone), invasive procedure recommendations were reduced from 57% (n = 154 cases out of 268 cases) to 18% (n = 44 cases out of 251 cases), a three-fold decrease (*p* < 0.001) [12]. The Percepta classifier was also evaluated prospectively in a large registry cohort, and in patients with a pre-test low/intermediate risk of malignancy for whom a nondiagnostic result would have led to a subsequent invasive procedure, 34.3% of patients were able to be down-classified. Of these down-classified patients, 73.9% had a change in their management plan from an invasive procedure to radiographic surveillance [13].

These studies did not address the impact on decision-making of the classifier when the result was a reclassification from high risk to very high risk. A second-generation classifier was later developed utilizing RNA whole transcriptome sequencing and machine learning. The Percepta Genomic Sequencing Classifier (Percepta GSC, Veracyte, Inc.) utilizes RNA sequencing of transcripts from 1232 genes, as well as clinical factors, to improve classifier performance with both down-classification and up-classification of pre-test risk of malignancy. Percepta GSC provides functionality in all three categories of pre-test risk, low (< 10%), intermediate (10–60%) and high (> 60%). It has the ability to risk re-stratify from low risk to very low risk of malignancy with an NPV of 100% and risk re-stratify from high risk to very high risk of malignancy with a positive predictive value (PPV) of 91.5% [14].

The ability of Percepta GSC to up-classify from a pretest high risk to very high risk of malignancy could significantly impact clinical decision-making following a nondiagnostic bronchoscopy by increasing confidence in the decision to proceed with an aggressive approach involving surgical diagnosis and cure. This could result in improved outcomes because of a shorter time to diagnosis and treatment [15] while reducing the number of interval procedures. The primary objective of this study was to determine the impact of a Percepta GSC up-classification from pre-test high-risk (> 60%) to post-test very high-risk (> 91%) in the initial management of suspicious pulmonary lesions. We hypothesized that up-classification by Percepta GSC will result in a recommendation for either surgical resection or other ablative therapy as recommended in the American College of Chest Physicians (ACCP) Guidelines, obviating the need for additional procedures in patients classified as being at high risk of malignancy [5].

## Methods

### Study overview

This was a prospective, case-randomized decision impact study designed to evaluate pulmonologists’ management recommendations in patients undergoing workup for lung cancer who had an inconclusive bronchoscopy to determine the clinical utility of Percepta GSC risk of malignancy reclassification from high to very high. The primary objective was to evaluate the frequency of change in the management of a lung nodule from additional diagnostic procedures to resection or ablative treatment with a Percepta very high result compared to no Percepta test. The secondary objective was to evaluate change in the level of confidence in the diagnosis in patients without Percepta testing to those with a Percepta-Very High result.

Institutional review board (IRB) review was obtained through a centralized IRB (Advarra IRB). The project was determined to be exempt from IRB oversight.

### Study participants

Study participants were U.S. board-certified, licensed, practicing pulmonologists from community, academic, and tertiary care centers. All participants had practiced pulmonary medicine for 40 years or less post-training and reported performing 10 or more bronchoscopies per month in patients suspected to have lung cancer. All study participants were required to correctly classify the risk of malignancy of two patient cases that had been adjudicated as high risk of malignancy (> 60%) by the initial treating physician and by a calculation of the Malignancy Risk Score (Mayo Clinic Model) [16].

### Patient population

Patient cases for this study were selected from the validation cohort (n = 412) for the Percepta GSC [13]. This cohort was derived from the Airway Epithelial Gene Expression in the Diagnosis of Lung Cancer (AEGIS) trials (246 patients: AEGIS-1 and − 2, NCT01309087 and NCT00746759) and the Percepta Registry (166 patients) [9, 17]. The AEGIS trials were prospective, multicenter observational studies in which current and former smokers with no prior history of cancer underwent a diagnostic bronchoscopy for suspected lung cancer. The design of these studies has been described elsewhere in detail [9, 13, 17]. The Percepta Registry was a multi-center prospective registry that included patients with lung nodules who underwent clinically indicated diagnostic bronchoscopy at 34 medical centers across the US. The nodules in the combined Percepta GSC cohort were separated into low (< 10%), intermediate (10–60%) or high (> 60%) pre-test risk of malignancy based upon either physician assessed risk or via a published risk assessment model. Approximately 35% of the nodules determined be high risk prior to the procedure. Twenty-seven percent of these cases were up-classified to very high risk by Percepta GSC with a 91.5% positive predictive value (PPV) of malignancy [13].

### Patient cases

Cases were extracted from the Percepta GSC validation cohort in which (1) the pre-bronchoscopy risk of malignancy was determined by the treating physician to be high-risk (pre-test risk of malignancy of > 60%) and was up-classified to very high risk (PPV 91.5%) by Percepta GSC, and (2) the initial bronchoscopy procedure was not diagnostic for cancer. Thirty-seven cases met both criteria. Case details were de-identified and extracted from case report forms and described in detailed vignettes. Each of these patient cases was further determined to be high risk (> 60%) by the Malignancy Risk Score (Mayo Clinic Model) [16].

The study involved two arms, an independent arm and a cross-over arm, in which 37 cases were each presented in three formats (Additional file 1: Figure 1) generating 117 unique case presentations in total. The cross-over arm used a pre–post format where the case was presented first without and then subsequently with the Percepta GSC result, with survey questions intervening (format A). In a second arm of the study (the independent arm), the cases were presented in two additional formats: (B) without the Percepta GSC result, and (C) with the Percepta GSC result. Patient cases in these three formats were then randomized. All surveys were administered by an external consulting company (Outcomes Insights).

### Data collection

Physicians were asked to provide basic information about their clinical practice including type of practice, bronchoscopy techniques used, and how they typically manage patients undergoing a diagnostic workup for suspected lung cancer. The validated performance characteristics and intended use of Percepta GSC was explained to each respondent, and a Percepta GSC test report with up-classification from high to very high risk was reviewed (Additional file 1: Figure 2). Physicians were required to demonstrate an understanding of the report before proceeding.

Each physician was presented with 10 de-identified patient cases in one of the three formats. Formats B and C (the independent arms) were matched in terms of number of reviews, and each case was reviewed a minimum of 26 times. The physician was informed that the nodule had been determined to be high risk (> 60% risk of malignancy) both by the initial treating physician using all of the information available to them and independently by the Malignancy Risk Score (Mayo Clinic Model). The survey taker was then provided with the clinical history, imaging findings, and cytology and pathology results from the nondiagnostic bronchoscopy. If appropriate for the format, the Percepta GSC result was included in the data for review or initially withheld and then provided, with interval questioning.

For each case, the physician was asked to estimate the patient’s risk of malignancy and provide their level of confidence in that estimate on a 7-point scale (1 = not at all confident; 4 = neutral; 7 = extremely confident) (Additional file 1: Figure 3). They were then asked to determine the next management step for that patient. Choices were (1) PET scan (diagnostic, not staging); (2) CT surveillance (at an option of 1, 3, or 6 months); (3) repeat biopsy (transthoracic biopsy or needle aspiration, or repeat bronchoscopy for transbronchial biopsy, transbronchial needle aspiration or endobronchial biopsy); (4) ablative therapy (surgery, stereotactic body radiotherapy or radiofrequency ablation; or (5) “other”. If the physician chose PET, they were informed that the PET yielded “indeterminate results (PET scan results did not significantly alter your assessment of malignancy risk for this patient)”. The physician was then prompted to make another management choice. They were next asked to rate their level of confidence in what they had ultimately recommended as the next step on the 7-point scale. If the case was in the pre–post format, the survey taker was asked to estimate risk of malignancy, determine the next step, and then assess their level of confidence without a Percepta GSC result. They were then given the Percepta result showing up-classification to very high risk, followed by a reassessment of the estimated risk of malignancy, the most appropriate next step, and level of confidence. Given that physician confidence is subjective, variable and highly individualized, we confined our analyses of confidence to the pre–post cohort in order to control for these factors, essentially isolating the effect of the GSC result by matching at the individual physician level.

### Data analysis

The three case formats resulted in 3 separate cohorts. In the pre–post arm, a comparison was made within the cohort, pre vs. post. In the independent arm, a comparison was made between the separate cohorts, without a GSC result vs. with a GSC result. Rates of definitive procedures were compared in terms of proportional estimates and hypothesis testing using binomial statistics. Additionally, to evaluate which clinical factor(s) had a significant effect on physicians’ decisions and whether the Percepta GSC test result remains as a significant effect when other clinical factors are controlled for, respondents’ decisions were analyzed using generalized linear models including those clinical factors. Univariate and multivariate regression models were used to assess the individual and combined impact of a Percepta result and clinical variables to the physician’s decision. All statistical tests were conducted at a significance level of 0.05. All analyses were performed using R, version 4.1.0.

## Results

One hundred and one survey takers provided a total of 1341 evaluations of the 37 patient cases across the three different cohorts. The demographics of the survey takers are described in Additional file 1: Table 1. They represented pulmonologists from all regions in the U.S. in a variety of practice settings. 56% were current Percepta GSC users, while 44% had not previously used this test. The demographics of Percepta GSC Users compared to non- users is shown in Additional file 1: Table 2. Users were more likely to be Interventional Pulmonologists (40% vs. 14%, *p* = 0.001) but had significantly fewer years in practice (11 vs. 16 years, *p* = 0.03). Patient demographics and nodule characteristics are presented in Table 1a, b, respectively.

**Table 1:** Demographics of subjects (a) and their nodule characteristics (b)

	N = 37
a	
*Patient characteristics*	
Cohort	
AEGIS I/II	30 (81%)
Percepta registry	7 (19%)
Age	
Years (median (IQR))	73 (68,77)
Race	
White	28 (76%)
Black	8 (22%)
Asian	1 (2.7%)
Sex	
Female	17 (46%)
Male	20 (54%)
Smoking status	
Current smoker	19 (51%)
Former smoker	18 (49%)
Tobacco pack years	
Years (median (IQR))	59 (40,82)
b	
*Nodule characteristics*	
Nodule size (mm)	
Median (IQR)	23 (18,25)
Nodule location	
Central	17 (46%)
Peripheral	20 (54%)
Lung cancer (histologic type)	
Malignant	35 (95%)
Non-small cell	30 (81%)
Adenocarcinoma	13 (35%)
Squamous	8 (22%)
Not specified/unknown	9 (24%)
Small cell (Limited stage)	2 (5%)
Unknown	3 (8%)
Benign	2 (5%)

As expected given that this study focused on nodules with a high pre-bronchoscopy risk of malignancy and up-classification by Percepta GSC to very high risk with PPV of 91%, the majority of the cases were malignant. Most of the nodules were relatively large, with a median size of 23 mm (Inter-quartile range (IQR), 18–25 mm). Patients had a median age of 73 years, were slightly more often male, and were balanced between current and former smokers, with a high median pack year smoking burden (median, 59 pack years, IQR 40–82 pack years).

### Percepta GSC up-classification had a marked impact on the decision to proceed to definitive intervention following a nondiagnostic bronchoscopy in both the independent and cross-over arms

After reviewing clinical and radiographic information for each case, survey takers recommended surgical resection or other ablative therapy as the next appropriate management step (after PET exclusion) in only 17% of cases in both the independent cohort without Percepta GSC and the first (pre-) half of pre–post cohort (Table 2).

**Table 2:** Outcome variables by cohort

Characteristic	Independent		Pre–post	
	Without percepta	With percepta	Without percepta	With percepta
	N = 330^a^	N = 337^a^	N = 341^a^	N = 341^a^
Next step				
Ablative therapy^b^	56 (17%)	150 (45%)	57 (17%)	191 (56%)
CT	98 (30%)	40 (12%)	81 (24%)	27 (7.9%)
Other	9 (2.7%)	1 (0.3%)	14 (4.1%)	3 (0.9%)
Repeat biopsy	167 (51%)	146 (43%)	189 (55%)	120 (35%)
CT interval				
1 month	7 (7.1%)	2 (5.0%)	5 (6.2%)	5 (19%)
3 months	82 (84%)	30 (75%)	66 (81%)	21 (78%)
6 months	9 (9.2%)	8 (20%)	10 (12%)	1 (3.7%)

^a^n (%)

^b^Includes surgery

**Fig. 1:**
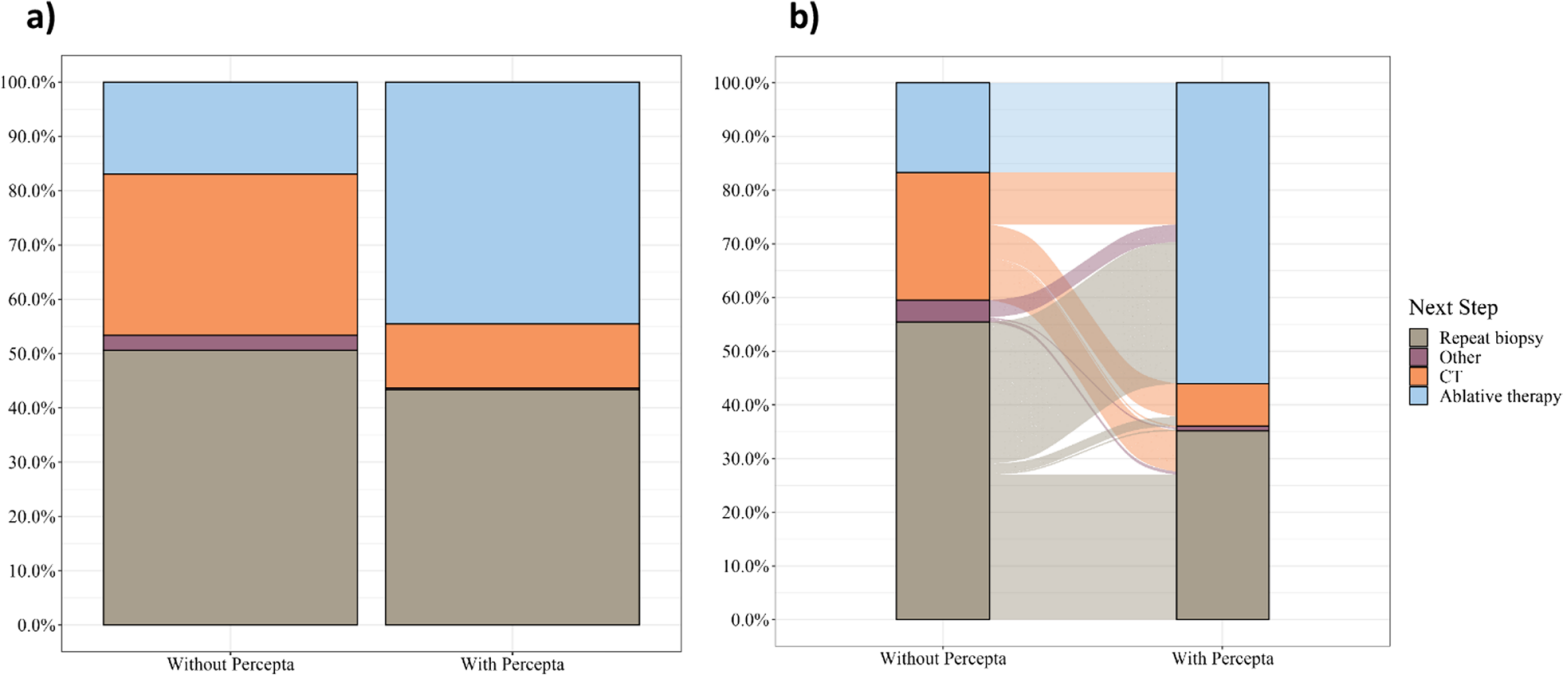
Recommended next step (PET excluded) in **a** the independent cohorts, and **b** the pre–post cohort. **a** Comparing two independent cohorts, there was a significantly higher rate of choice of surgical resection or other ablative therapy with a Percepta GSC very high risk result (1st column) compared to the cohort without a Percepta GSC very high risk result (2nd column) (*p* < 0.0001). **b** In the pre–post cohort, providing a Percepta GSC very high risk result significantly increased the rate of choice of surgical resection or other ablative therapy *p* < 0.0001)

In the independent cohort where a Percepta GSC result was received, the rate of recommendation for surgical resection was significantly higher at 45% (150/337) compared to the independent cohort without a GSC result (Odds Ratio 3.81, 95% CI 2.19–6.79, *p* < 0.001) (Table 2, Fig. 1a). In the pre–post cross-over cohort, the rate increased from 17 to 56% (191/341) of cases following the review of the GSC result (Odds Ratio 12.1, 95% CI 6.25–24.9, *p* < 0.001) (Table 2, Fig. 1b). In both instances where a GSC result was available, physicians were less likely to recommend an interval CT scan and less likely to recommend repeat biopsy. In the pre-post cohort, Percepta users were more likely to choose ablative therapy than non-Percepta users when given Percepta GSC results, though the difference was not statistically significant (61–52%, *p* value = 0.10).

### Percepta GSC up-classification increases pulmonologists’ confidence in decision-making following a nondiagnostic bronchoscopy

**Fig. 2:**
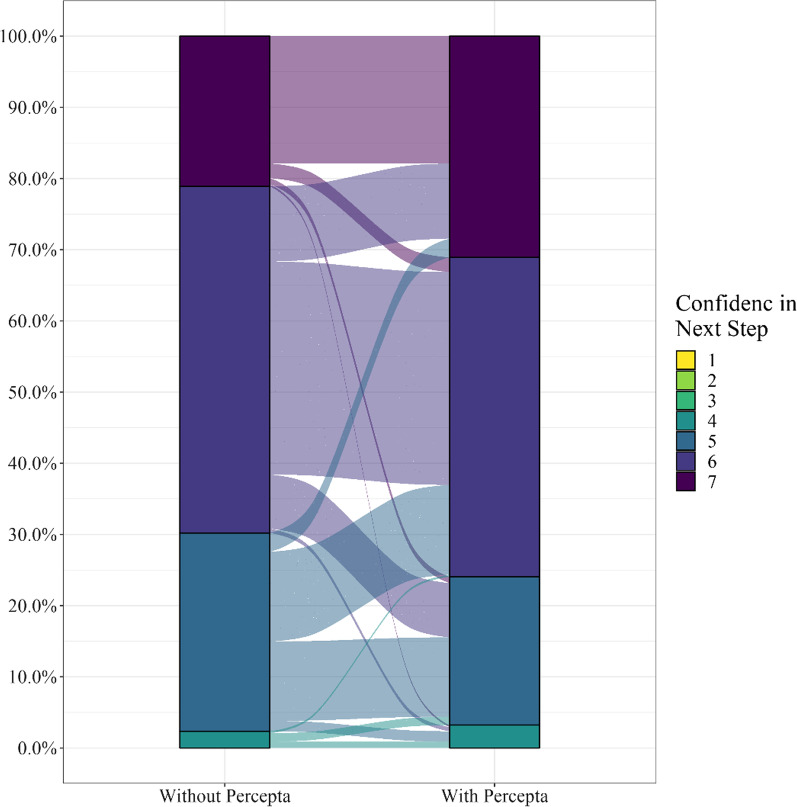
Sankey plot showing physician confidence levels in the pre–post cohort. There was a significant increase in physician confidence in the recommended next step following a review of a Percepta GSC very high risk result in the pre–post cohort (*p* = 0.0017)

Overall, baseline physician confidence in the management plan was very high without a Percepta GSC result, with 70% of survey takers rating their level of confidence at 6 or 7 on the 7-point scale. Receipt of a Percepta GSC very high risk result further increased confidence, with 76% of survey takers rating their level of confidence at 6 or 7. There was a statistically significant increase from 21 to 31% (*p* = 0.0017) of cases where the respondent rated their confidence at a level of 7 (Fig. 2).

There were 44 individual instances where confidence in the treatment plan actually decreased following a GSC result. Comparing those 44 instances to the larger number of instances (n = 297) where confidence increased, we observed no significant demographic differences (Additional file 1: Table 4). The decrease in confidence in this subset was not specific to any case or any individual physician. A drop in confidence was seen at least once in 33 different physicians in 27 different patients. Of the 33 different physicians, 32 physicians (97%) were shown at least one other pre-post case where Percepta GSC increased their confidence. In 42 of 44 (95%) of instances where confidence in the treatment plan went down, respondents chose an initial plan for either CT or repeat biopsy. Of those 42, 30 (71%) maintained a choice of either CT or repeat biopsy but had decreased confidence in that decision in light of the GSC result.

### Baseline physician risk assessment is suboptimal but improves with percepta GSC

**Fig. 3:**
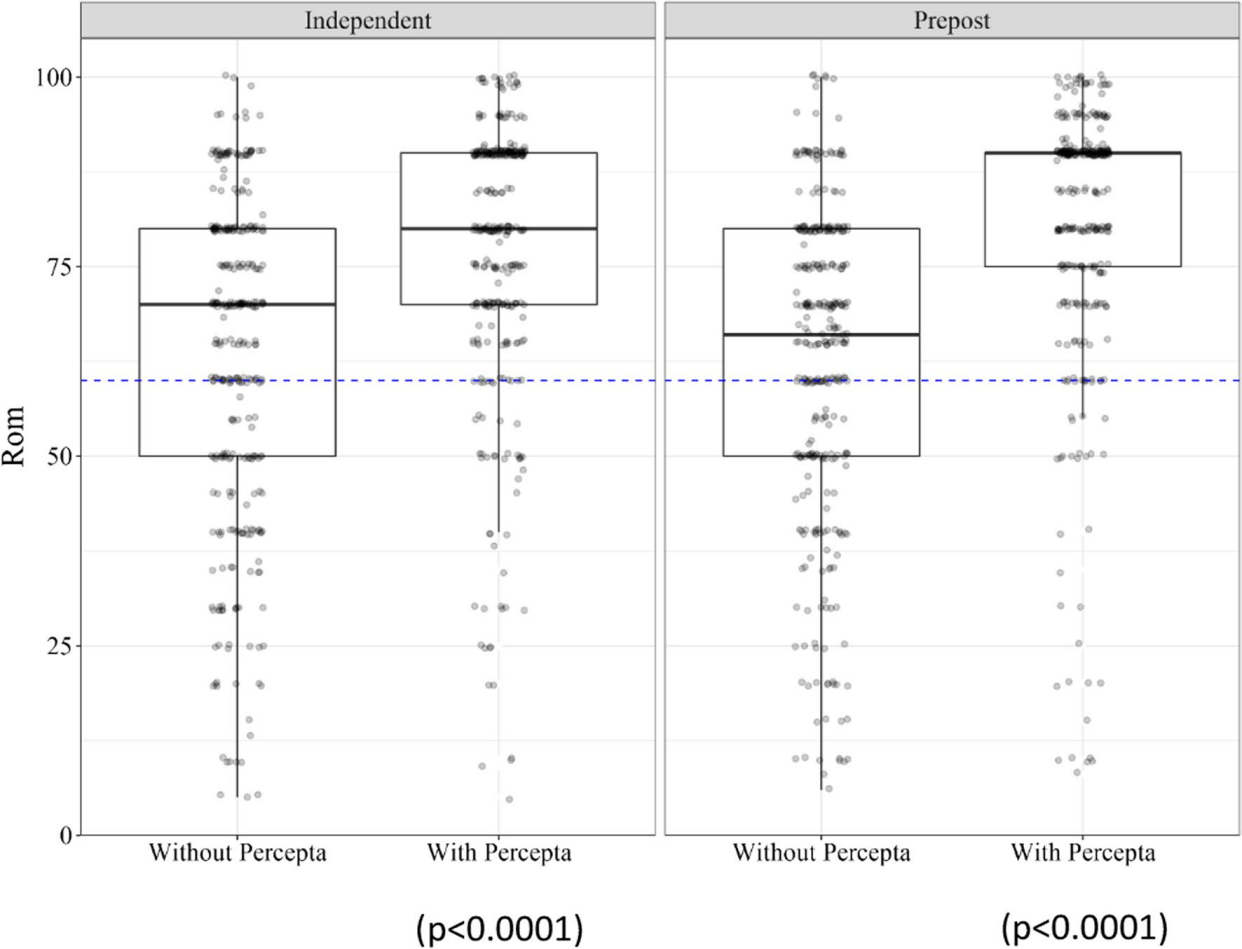
Distribution of physician survey taker assessment of the risk of malignancy. A Percepta GSC very high risk up-classification resulted in a signficantly higher mean assessment of ROM in the independent cohort with Percepta GSC compared to the independent cohort without a GSC result (left). Providing a very high risk result significantly increased the mean assessment of ROM in the pre–post cohort (right). In all three cohorts, physicians frequently assessed ROM to be < 60%. *ROM* risk of malignancy

Survey takers were asked to submit their assessment of the risk of malignancy in each case. In each instance, they were aware that the bronchoscopist had determined that the risk of malignancy exceeded 60% and that the Mayo Clinical Risk Model agreed. Yet in 33% of cases in both the independent cohort without a GSC result and in the pre–post cohort prior to a GSC result, the surveyed physician assessed the risk of malignancy to be less than 60% (Additional file 1: Table 3). Given that 95% of the cases reviewed were, in fact, malignant (Table 1b) this represents a notable underappreciation of the risk of cancer in these cases. Figure 3 shows the distributions of the assessed risk in each of the cohorts.

**Fig. 4:**
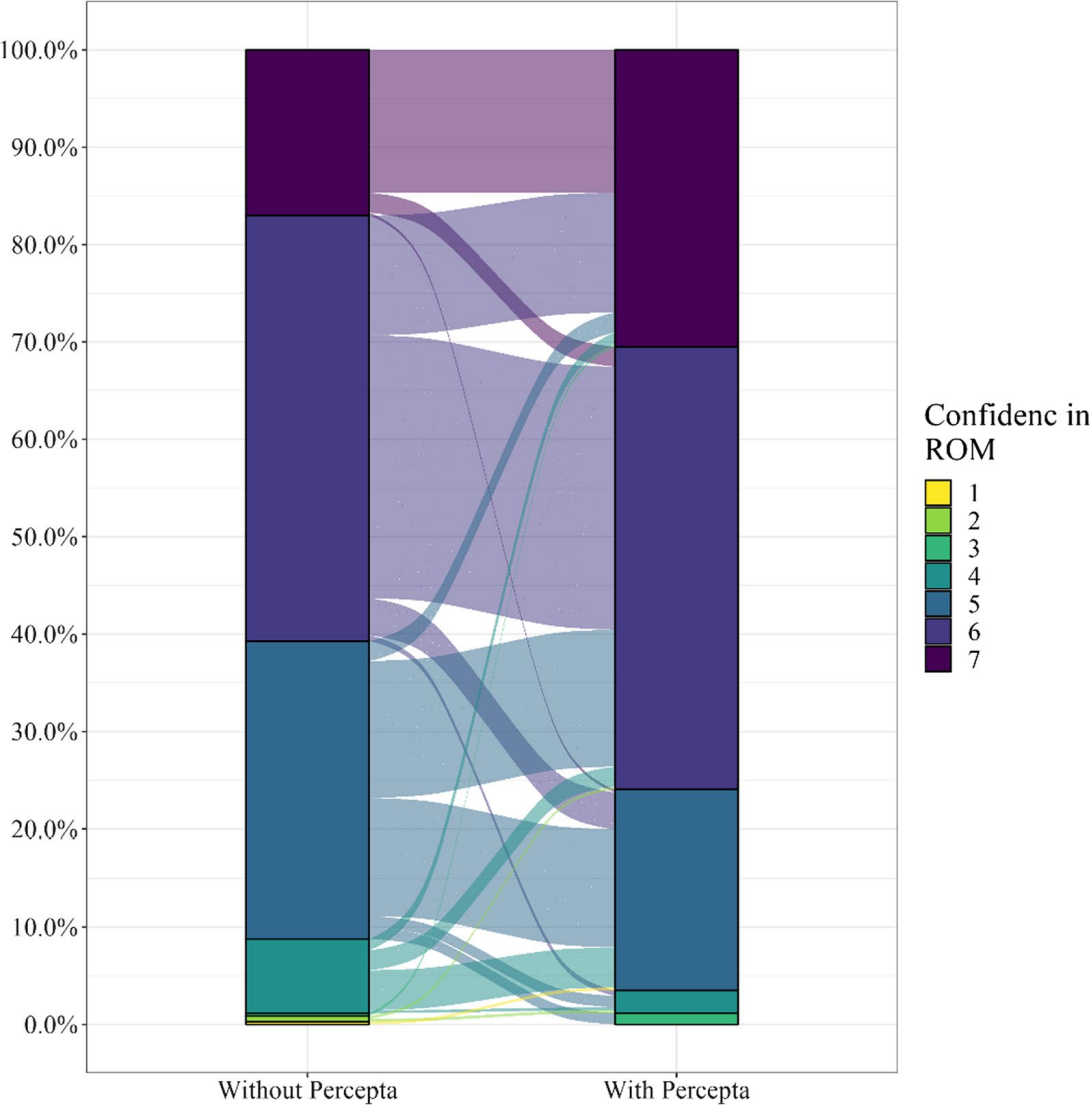
Physician confidence in the assessment of the risk of malignancy (ROM) in the pre-post cohort. Following a review of a Percepta GSC very high risk result, physician confidence in the assessment of ROM increased significantly (*p* = 0.0017)

The independent cohort with a Percepta GSC result had a significantly higher mean assessed risk of malignancy than the independent cohort without (*p* < 0.0001), and the mean risk of malignancy assessed in the pre- and post- cohort increased significantly following review of the results (*p* < 0.0001). Measured in the pre- and post-cohort to control for variability in physicians’ baseline levels of confidence, confidence in the assessment of the risk of malignancy also significantly increased following a Percepta GSC very high risk result (*p* < 0.001) (Fig. 4).

## Discussion

Nondiagnostic bronchoscopies are common in the management of indeterminant pulmonary nodules, including those at high pre-bronchoscopy risk of malignancy, and decision-making in these patients can be difficult. Our data shows that a very high risk Percepta GSC result, with its PPV > 91%, increases the frequency with which Pulmonologists recommend proceeding directly to surgical resection or other ablative therapy rather than a repeat biopsy or additional CT imaging, aligning with current ACCP guidelines for high risk nodules. For patients with early stage lung cancer, this is likely to result in a reduction in the time to appropriate therapy, better outcomes[15], and reduced health care costs [3]. Moreover, a Percepta GSC very high risk result increases physician confidence in this recommendation and their confidence in their assessment of a patient’s risk of malignancy. The ability to up-classify risk could also increase confidence in a physician’s decision to proceed directly to non-invasive ablative therapy in selected patients who are at prohibitive risk of complications from either surgery or transthoracic needle sampling.

Our study has some limitations that merit discussion. First, this was a decision-impact study, not Percepta GSC being used in a real-world setting, although we sought to closely model this with the pre–post format. There are several ways in which our survey format differed from real-world use. Although our survey takers had access to clinical data, including detailed radiology reports, a pulmonologist would typically review the images themselves and may have come to a different determination of risk with that data. To minimize the impact of this, all CTs were interpreted by expert thoracic radiologists and all salient features were described in detail.

This decision impact study focused narrowly on the capacity of Percepta GSC to up-classify risk in certain patients from high to very high. Limiting the GSC results in this way may have biased the survey takers’ responses toward more aggressive treatment choices, and therefore results of this study might not be fully representative of physician behavior in a real-world setting where the classifier may be used in all risk-groups, not just high-risk.

Many pulmonologists utilize PET imaging in the risk assessment of indeterminate nodules [18]. Given that our intent was to specifically assess use of invasive procedures, survey takers who chose PET as a first option were given an indeterminant result and asked to choose a second option. Although this was imposed, this is not an uncommon clinical scenario. This was intended to maximize diagnostic uncertainty to model real-world decision-making and to avoid confounding the primary measured outcome.

In an actual patient case, a Percepta GSC result would be returned approximately 7 days after the bronchoscopy, and the data would not be evaluated at the same time as, or immediately following a review of a case with a nondiagnostic pathology result. Our pre–post cohort most closely resembles the sequential decision-making of a real-life bronchoscopy, and it is in this cohort where we saw our largest impact on the recommendation for ablative therapy. Finally, our survey did not include the important factor of patient preference. Shared decision-making and incorporation of an individual patient’s perspectives and values is increasingly recognized as a vital part of managing pulmonary nodules, particularly those with a high risk of malignancy [19]. However, the additional information provided by a Percepta GSC up classification to very high risk could provide reassurance to patients as well their health care providers, allowing for a more informed and confident decision.

It is worth noting that while the original bronchoscopist in each case and the Mayo Risk Model called all 37 cases high-risk, in many instances our survey takers did not. That reflects the wide variability in physicians’ ability to accurately assess the risk of malignancy in indeterminant lung lesions. The fact that the Percepta GSC result improved the assessment is evidence that better, more objective tools are needed for this task.

Our study has a number of important strengths. While the prior decision impact study of the Percepta bronchial genomic classifier focused on intermediate risk nodules and the utility of a down-classification of risk by a Percepta result, the current study addresses decision-making in nodules at high risk of malignancy. Arguably, decision-making in these cases following a nondiagnostic bronchoscopy is more difficult, given the potential for morbidity or even mortality from complications of additional invasive procedures or surgery. Our study used actual cases from the AEGIS trials used to develop the original classifier and from the Percepta Registry, so our survey takers were being tested on Percepta GSC’s intended use population. All of the cases had a genuine nondiagnostic bronchoscopy, and all had a real Percepta GSC result. Our study involved pulmonologists with a broad range of experience, across diverse practice settings and geographical location, and physicians who had not previously used Percepta GSC were well-represented. Finally, this study looked at decision-impact using two different models for presenting data to the survey takers, increasing the validity of the strong findings for the primary endpoint.

## Conclusions

The Percepta GSC very high risk reclassification resulted in an eightfold increase in the frequency of a recommendation to proceed to curative therapy following a nondiagnostic bronchoscopy. Combined with the classifier’s capacity to down classify risk in patients with benign lesions [13] (Additional file 1: Figure 4), Percepta GSC has the potential to guide physician decision-making in the full spectrum of patients with indeterminant pulmonary lesions and a nondiagnostic bronchoscopy. Use of the classifier will allow more patients with early lung cancer to proceed more quickly to potentially curative therapy without additional intervening procedures, while helping more patients with benign disease avoid further unnecessary procedures.

## Supplementary Information

**Additional file 1**. Supplementary figures and tables.

## Data Availability

The datasets generated during the current study are not publicly available due to concerns regarding participant confidentiality and proprietary information but are available upon reasonable request from the corresponding author.

## Abbreviations

GSC: Genomic sequencing classifier
AEGIS: Airway Epithelial Gene Expression in the Diagnosis of Lung Cancer
PPV: Positive predictive value
AUC: Area under the curve
NPV: Negative predictive value
ACCP: American College of Chest Physicians
IRB: Institutional review board
IQR: Inter-quartile range
ROM: Risk of malignancy
