# Supplementary material for: Percepta Genomic Sequencing Classifier and decision-making in patients with high-risk lung nodules: a decision impact study
Source: BMC Pulm Med. 2022 Jan 6;22:26. doi: 10.1186/s12890-021-01772-4 (PMC8740045; doi:10.1186/s12890-021-01772-4)
Supplement: Supplementary file 1 — Additional file 1. Supplementary figures and tables. [file 12890_2021_1772_MOESM1_ESM.docx]

**Supplementary Materials**

1. Table S1. Demographics of physician survey-takers
2. Table S2. Frequency with which physicians assessed the risk of malignancy to be less than 60%
3. Table S3. Frequency of physicians estimating a risk of malignancy ≤ 60% without and with a Percepta GSC very high risk result.
4. Table S4. Demographics of physicians reporting an increase in confidence in the treatment plan compared to those reporting a decrease in confidence following a Percepta GSC result.
5. Figure S1. Allocation of cases to physician survey takers
6. Figure S2. Percepta GSC risk re-stratification algorithm
7. Figure S3. Percepta GSC result report for risk re-stratification high risk to very high risk
8. Figure S4. Confidence scale

**Table S1.** Demographics of survey takers.

| **Survey Taker Characteristics** | **N=101^1^** |
| --- | --- |
| Use of Percepta in medical practice |  |
| Yes | 57 (56%) |
| No | 44 (44%) |
| Medical Specialty |  |
| Interventional Pulmonology | 29 (29%) |
| Pulmonary Critical Care | 29 (29%) |
| Pulmonology | 43 (43%) |
| Length of time in medical practice (years) | 13 (8, 23) |
| Region of medical practice (within the US) |  |
| Midwest | 29 (29%) |
| Northeast | 29 (29%) |
| South | 29 (29%) |
| West | 14 (14%) |
| Medical practice setting |  |
| Academic or Teaching Hospital | 50 (50%) |
| Community-Based | 51 (50%) |
| Bronchoscopies performed for suspect lung cancer (monthly) | 15 (10, 25) |
|  |  |

^1^n (%); Median (IQR, interquartile range)

**Table S2.** Demographics of survey takers, Percepta GSC Users vs. Non-Users.

| **Characteristic** | **Non-User**, N = 44*^1^* | **User**, N = 57*^1^* | **p-value***^2^* |
| --- | --- | --- | --- |
| Specialty |  |  | 0.001 |
| Interventional Pulmonology | 6 (14%) | 23 (40%) |  |
| Pulmonary Critical Care | 11 (25%) | 18 (32%) |  |
| Pulmonology | 27 (61%) | 16 (28%) |  |
| Years of Practice | 16 (11, 24) | 11 (5, 21) | 0.03 |
| Region |  |  | 0.2 |
| Midwest | 14 (32%) | 15 (26%) |  |
| Northeast | 15 (34%) | 14 (25%) |  |
| South | 8 (18%) | 21 (37%) |  |
| West | 7 (16%) | 7 (12%) |  |
| Type of Practice |  |  | 0.055 |
| Academic or Teaching Hospital | 17 (39%) | 33 (58%) |  |
| Community-Based | 27 (61%) | 24 (42%) |  |
| Bronchscopies Per Month | 15 (12, 31) | 15 (10, 25) | 0.069 |
| *^1^*n (%); Median (IQR) *^2^*Pearson's Chi-squared test; Wilcoxon rank sum test | | | |

**Table S3.** Frequency of physicians estimating a risk of malignancy ≤ 60% without and with a Percepta GSC very high risk result.

| **<=60% ROM** | **Without** | **With** |
| --- | --- | --- |
|  | **Percepta** | **Percepta** |
| Independent | 33% | 12.50% |
| Pre/post | 32.80% | 8.80% |

ROM = risk of malignancy

**Table S4.** Demographics of physicians reporting an increase in confidence in the treatment plan compared to those reporting a decrease in confidence following a Percepta GSC result.

|  | **Same/More Confident after** | **Less Confident after** | **P-value^2^** |
| --- | --- | --- | --- |
|  | **Percepta GSC** | **Percepta GSC** |  |
|  | (N=297)^1^ | (N = 44)^1^ |  |
| Percepta User |  |  | 0.2 |
| Non-User | 133 (45%) | 24 (55%) |  |
| User | 164 (55%) | 20 (45%) |  |
| Specialty |  |  | 0.6 |
| Interventional Pulmonology | 83 (28%) | 15 (34%) |  |
| Pulmonary Critical Care | 83 (28%) | 13 (30%) |  |
| Pulmonology | 131 (44%) | 16 (36%) |  |
| Years of Practice | 13 (9, 23) | 12 (8, 20) | 0.5 |
| Type of Practice |  |  | 0.7 |
| Academic or Teaching Hospital | 137 (46%) | 19 (43%) |  |
| Community-Based | 160 (54%) | 25 (57%) |  |
| Bronchoscopies Per Month | 15 (10, 25) | 15 (10, 30) | 0.5 |
| ^1^ n (%); Median (IQR) |  |  |  |
| ^2^ Pearson's Chi-squared test; Wilcoxon rank sum test | |  |  |

**Figure S1.** Allocation of cases to physician survey takers. (Not all physicians received cases in all three formats.)


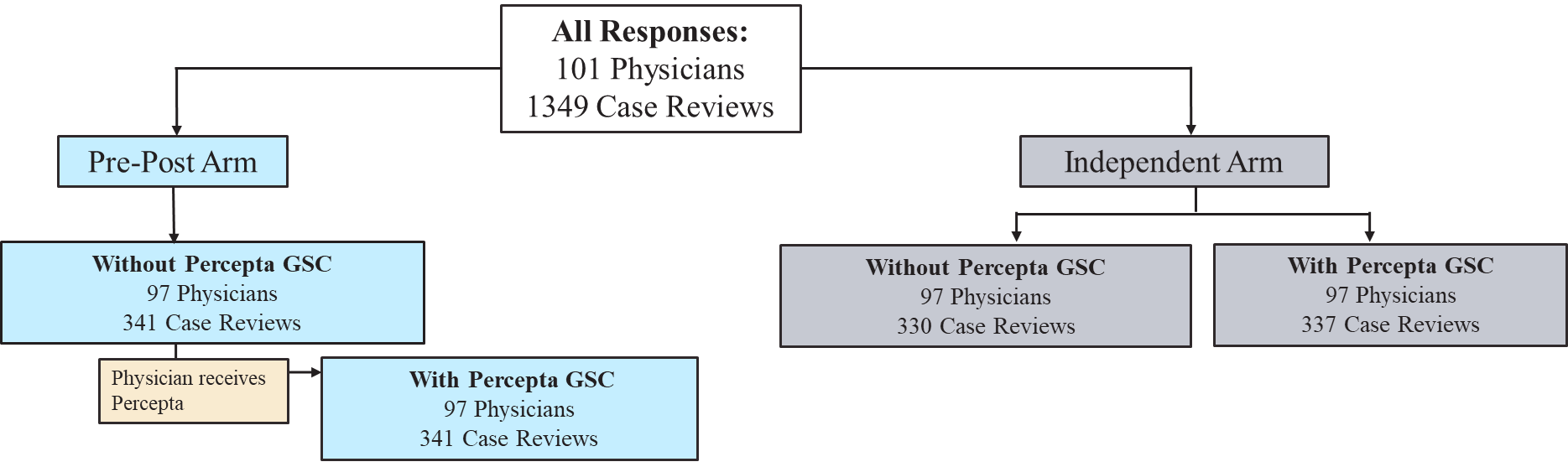


**Figure S2.** Percepta GSC result report for risk re-stratification high risk to very high risk.

**Figure S3.** Confidence scale for assessment of physician confidence.

| Not at all Confident |  |  | Neutral |  |  | Extremely Confident |
| --- | --- | --- | --- | --- | --- | --- |
| 1 | 2 | 3 | 4 | 5 | 6 | 7 |

**Figure S4.** Potential risk re-stratification algorithm for Percepta GSC.**
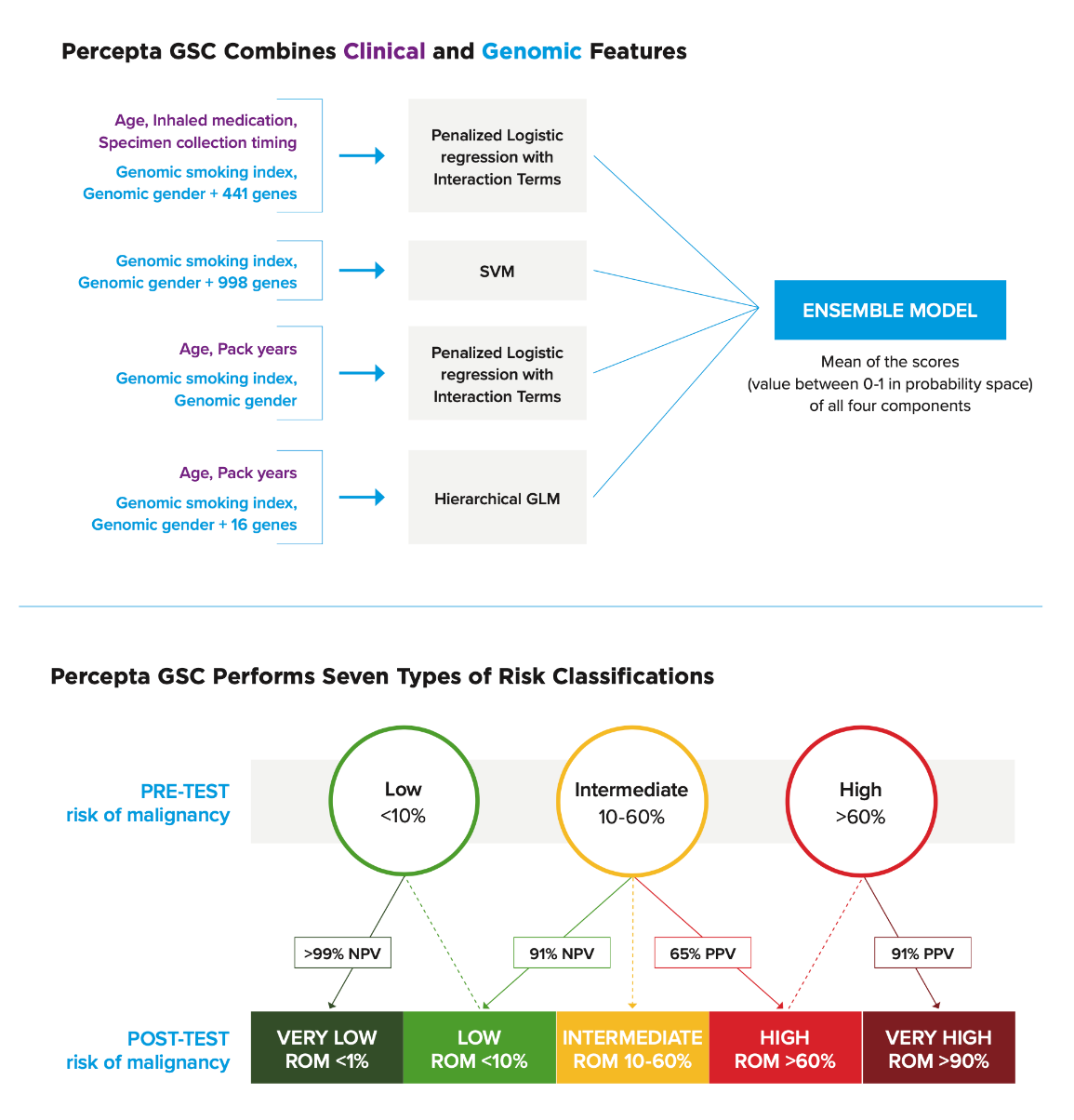
**

PPV=positive predictive value; NPV=negative predictive value; ROM=risk of malignancy
